# Non-psychotropic phytocannabinoid interactions with voltage-gated sodium channels: An update on cannabidiol and cannabigerol

**DOI:** 10.3389/fphys.2022.1066455

**Published:** 2022-11-10

**Authors:** Mohammad-Reza Ghovanloo, Sulayman D. Dib-Hajj, Samuel J. Goodchild, Peter C. Ruben, Stephen G. Waxman

**Affiliations:** ^1^ Department of Neurology, Yale University School of Medicine, New Haven, CT, United States; ^2^ Department of Cellular and Molecular Biology, Xenon Pharmaceuticals Inc., Burnaby, BC, Canada; ^3^ Department of Biomedical Physiology and Kinesiology, Simon Fraser University, Burnaby, BC, Canada

**Keywords:** cannabidiol (CBD), cannabigerol (CBG), voltage-gated sodium (Nav) channels, excitability, pharmacology

## Abstract

Phytocannabinoids, found in the plant, *Cannabis sativa*, are an important class of natural compounds with physiological effects. These compounds can be generally divided into two classes: psychoactive and non-psychoactive. Those which do not impart psychoactivity are assumed to predominantly function *via* endocannabinoid receptor (CB) -independent pathways and molecular targets, including other receptors and ion channels. Among these targets, the voltage-gated sodium (Nav) channels are particularly interesting due to their well-established role in electrical signalling in the nervous system. The interactions between the main non-psychoactive phytocannabinoid, cannabidiol (CBD), and Nav channels were studied in detail. In addition to CBD, cannabigerol (CBG), is another non-psychoactive molecule implicated as a potential therapeutic for several conditions, including pain *via* interactions with Nav channels. In this mini review, we provide an update on the interactions of Nav channels with CBD and CBG.

## Introduction

The cannabis plant contains over 120 active phytocannabinoids (Morales et al., 2017). Among these molecules, there are some that are psychotropic, and others that are not. Cannabidiol (CBD) is the primary non-psychotropic phytocannabinoid (Pertwee, 2008; Ghovanloo and Ruben, 2021). CBD has received extensive attention in recent years due to many anecdotal and some clinically substantiated reports of efficacy against various conditions (Billakota et al., 2019; Ghovanloo and Ruben, 2021). The interest in CBD has increased since the success of Epidiolex (therapeutic CBD) in large-scale clinical trials against Dravet and Lennox-Gastaut syndromes, which are severe pediatric-onset epileptic encephalopathies (Devinsky et al., 2017; Devinsky et al., 2018). However, despite its clinical efficacy, the exact mechanism of action for CBD remains undetermined.

In contrast to CBD, the main psychotropic phytocannabinoid, ∆9-tetrahydrocannabinol (THC), has a relatively clearcut mode of action. THC is a potent agonist at the human endocannabinoid (CB) receptors (∼13–90 nM) (Turner et al., 2017). The physiological function of these receptors is to respond to endogenous lipid agonists, anandamide and 2-arachidonoylglycerol (Devane et al., 1988; Devane et al., 1992; Vogel et al., 1993; Howlett, 2005; Di Marzo, 2008). Similar to CBD, THC has been shown to possess anticonvulsant properties in animal models; however, the noted psychotropic effects of this compound make it a less than ideal therapeutic candidate (Lynch et al., 2004; Pinsger et al., 2006; Russo and Guy, 2006; Mechoulam et al., 2007; Skrabek et al., 2008; Johnson et al., 2010; Ware et al., 2010).

CBD has low affinity for the CB receptors, where it has mild antagonistic effects (Tham et al., 2019). Therefore, CB-independent targets are the most likely molecular mechanisms underlying CBD’s efficacy. CBD was shown to interact with GPR55 receptors (Sylantyev et al., 2013; Kaplan et al., 2017), which are proteins that are expressed in excitatory and inhibitory synapses and which modulate synaptic plasticity. CBD is also a modulator of several TRP channels (De Petrocellis et al., 2011; De Petrocellis et al., 2012; Hassan et al., 2014; Pumroy et al., 2019), 5-HT1A receptors (Campos and Guimarães, 2008; Fogaça et al., 2014; Marinho et al., 2015), and an inhibitor of adenosine reuptake by voltage-dependent anion channel 1 (Rimmerman et al., 2013). Importantly, CBD is an inhibitor of voltage-dependent sodium (Nav) channels (Ghovanloo et al., 2018; Ghovanloo and Ruben, 2021; Zhang and Bean, 2021), some potassium channels (e.g., Kv2.1) (Ghovanloo et al., 2018; Orvos et al., 2020), calcium channels (Ross et al., 2008) and, in contrast, an activator of Kv7 channels in the nanomolar range (Zhang et al., 2022). Additionally, CBD directly modulates the biophysical properties of the bio-membrane itself (Ghovanloo et al., 2021; Ghovanloo et al., 2022b; Guard et al., 2022), which may facilitate an allosteric modulation of membrane proteins including, but not limited to, those noted above.

Among the CBD targets, the family of Nav channels are particularly interesting for three reasons. First, Dravet syndrome, the most notable condition for which CBD is efficacious, is commonly linked to genetic mutations in Nav1.1 (Yu et al., 2006; Kalume et al., 2007, 2015; Dravet, 2011; Devinsky et al., 2014; Peters et al., 2016; Gorman et al., 2021; Fouda et al., 2022). Nav1.1 is a key regulator of excitability in inhibitory circuits within the central nervous system. Second, CBD is reputed to have therapeutic value, substantiated by preclinical and animal studies, for a variety of excitability related disorders including pain, seizures, muscular problems, and arrhythmias, among others (Wade et al., 2004; Iannotti et al., 2019; Ghovanloo and Ruben, 2021). Dysfunction of various Nav channels in different tissues could trigger any of the noted conditions (Ghovanloo et al., 2016; Fouda et al., 2022). Third, amphiphilic compounds (e.g., Triton X-100) (Lundbæk et al., 2010) that modulate membrane elasticity (with properties that are similar to CBD) have been shown to allosterically stabilize Nav channel inactivation (Lundbæk, 2005; Lundbæk et al., 2010; Ghovanloo et al., 2021; Ghovanloo et al., 2022b). These reasons prompted us and others to study effects of CBD on Nav channels in detail over the past several years. CBD is now established as an effective Nav channel inhibitor (Ghovanloo et al., 2018; Fouda et al., 2020; Sait et al., 2020; Ghovanloo et al., 2021; Ghovanloo and Ruben, 2021; Zhang and Bean, 2021). Furthermore, these investigations suggested Nav channels are a promising pathway for cannabinoid-mediated reductions in macro excitability, with a substantial therapeutic potential. This pathway could be explored not just with CBD, but also with other compounds with similar physicochemical properties.

A common precursor for THC and CBD is cannabigerol (CBG) (Nachnani et al., 2021). Like THC (ChEMBL-calculated-LogD = 5.94) and CBD (ChEMBL-calculated-LogD = 6.60), CBG (ChEMBL-calculated-LogD = 7.04) is also a highly hydrophobic compound. Although CBG is less well studied than THC or CBD, the existing literature suggests that CBG’s pharmacological profile falls in between these two cannabinoids. Importantly, while CBG’s affinity for CB receptors is higher than CBD, CBG is non-psychotropic (Nachnani et al., 2021). This suggests that CBG could work through both CB-dependent and CB-independent (e.g., Nav channels, TRP channels, etc.) pathways without THC’s unwanted psychoactive effects (Muller et al., 2019; Ghovanloo et al., 2022a). With this combination of properties, CBG offers the potential to be a superior therapeutic compound than either CBD or THC. A comparison of the key targets between CBD and CBG is provided in Table 1.

**TABLE 1:** Comparison of a list of key receptors and ion channel targets between CBD and CBG. See (Almeida and Devi, 2020; Ghovanloo and Ruben, 2021; Nachnani et al., 2021) for more extensive reviews of these targets.

Target	CBD	CBG	References
CB1	Inverse agonist/antagonist	Weak agonist	(Cascio et al., 2010; Pollastro et al., 2011; Muller et al., 2019)
CB2	Inverse agonist	Partial agonist	(Cascio et al., 2010; Pollastro et al., 2011; Muller et al., 2019)
GPR55	Antagonist	Unknown	(Kaplan et al., 2017; Harding et al., 2018)
Nav	Inhibitor	Inhibitor	(Ghovanloo et al., 2018; Sait et al., 2020; Ghovanloo et al., 2021; Zhang and Bean, 2021; Ghovanloo et al., 2022a)
TRPA1	Agonist	Agonist	(De Petrocellis et al., 2011; Pollastro et al., 2011; Muller et al., 2019)
TRPV1	Agonist	Agonist	(De Petrocellis et al., 2011; Pollastro et al., 2011; Muller et al., 2019)
TRPV2	Agonist	Agonist	(De Petrocellis et al., 2011; Pollastro et al., 2011; Muller et al., 2019)
TRPV3	Agonist	Agonist	Muller et al. (2019)
TRPV4	Agonist	Agonist	Muller et al. (2019)
TRPM8	Antagonist	Antagonist	(De Petrocellis et al., 2011; Pollastro et al., 2011; Muller et al., 2019)
Kv7	Potentiator	Unknown	Zhang et al. (2022)
Kv2.1	Inhibitor	Unknown	Ghovanloo et al. (2018)
Cav	Inhibitor	Inhibitor	(Ross et al., 2008; Mirlohi et al., 2022)
Biomembrane	Modulator	Unknown	Ghovanloo et al. (2021)

Much of the molecular details of CBD’s interactions with Nav channels is reviewed in Ghovanloo and Ruben, 2021. In this short report, we provide new important updates on Nav channel mediated-cannabinoid pathway with a focus on CBD and CBG.

## Cannabidiol—Mechanism of action on sodium channels

We previously found that CBD is a non-selective Nav channel inhibitor. Using voltage-clamp experiments, we found that CBD inhibits all human Nav1.1-7 from the inactivated states, with potencies ranging from 1.9 to 3.8 µM, and steep Hill slopes of ∼3. We also found that CBD imparts similar effects on Nav gating: inhibiting G_max_ without changing voltage-dependence of activation, but hyperpolarizing steady-state inactivation and slowing recovery from inactivation (Ghovanloo et al., 2018). Furthermore, we found that when Nav channels enter deeper inactivated states, CBD slows the recovery kinetics even further, consistent with state-dependent Nav channel inhibition. CBD has an approximately 10-fold state-dependence, which makes it a moderately state-dependent Nav channel inhibitor (Ghovanloo et al., 2018; Ghovanloo and Ruben, 2021). From a molecular perspective, it may be theoretically conceivable to use CBD against Nav channelopathies that greatly impair inactivation (Ghovanloo et al., 2020).

**FIGURE 1:**
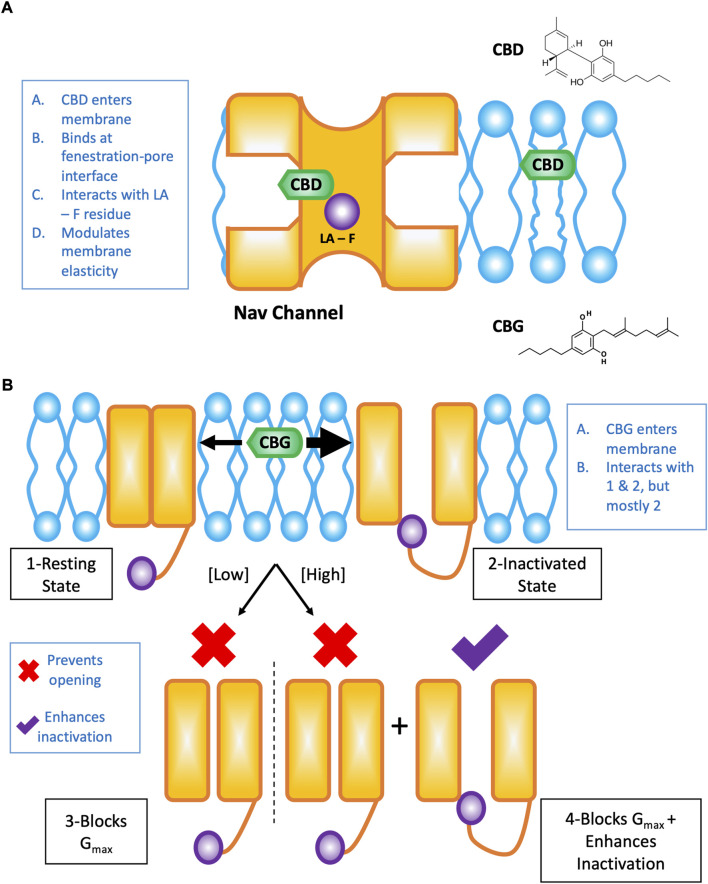
Cartoon summary of CBD and CBG effects on Nav channels. This figure is a redrawn version of our previously published papers (Ghovanloo and Ruben, 2021; Ghovanloo et al., 2022a). **(A)** Shows the pathway of CBD from the lipid phase through the mammalian Nav fenestration and into the pore, where it interacts to some extent by the local anesthetic (LA) site with the pore phenylalanine (F) residue. **(B)** Cartoon representation of the concentration-dependent modality of CBG effects on Nav channels. CBG, like CBD is a state-dependent Nav inhibitor with an increased affinity for the inactivated state. Overall, however, CBG causes a reduction in total conductance/channel opening more potently than inactivation is stabilized in the presence of CBG.

The effects of CBD on Nav channels are the result of interactions at the interface of the channel pore and fenestrations in which CBD directly blocks the pore (in part *via* the local anesthetic phenylalanine), and alterations to the membrane elasticity which indirectly stabilizes Nav channel inactivation (Figure 1A). These results were elucidated using structural- (Sait et al., 2020), functional- (Ghovanloo et al., 2018, 2021), and molecular dynamics simulation-based (Ghovanloo et al., 2021) studies. It is important to note that the drug pathway from the membrane phase and through the Nav channel fenestrations is pharmacologically important, and has been elucidated with various drugs previously (Hille, 1977; Gamal El-Din et al., 2018).

One of CBD’s main proposed clinical application is in pain treatment (Ward et al., 2014). There are several different Nav channels within the peripheral sensory pathway (Rush et al., 2007; Dib-Hajj et al., 2010), which include both tetrodotoxin-sensitive (TTX-S) and resistant (TTX-R) (Nav1.8/9) subtypes (Cummins et al., 2007; Bennett et al., 2019). In contrast to most other Nav channels, the TTX-R channels have a hyperpolarized voltage-dependence of slow inactivation relative to their fast inactivation and a more rapid entry into slow inactivation than other Nav channels (Vilin and Ruben, 2001; Blair and Bean, 2003; Ghovanloo et al., 2016). Therefore, the slow inactivation properties of these channels can limit repetitive firing in their native environment. Because of these properties, drugs that target slow inactivated states could be effective for reducing repetitive firing in sensory neurons.

A recent study determined that CBD at 500 nM has tight binding to the slow inactivated states of Nav1.8 (Zhang and Bean, 2021). These low concentrations of CBD had little effect on the first several action potentials, but as the current injection became larger, CBD reduced firing. Furthermore, CBD reduced the action potential height, widened the action potential, reduced afterhyperpolarization, and increased the propensity of entering depolarization block (Zhang and Bean, 2021).

Another recent study has shown that CBD-dominant nutraceutical products can inhibit Nav channels even more potently than pure CBD (difference is in the order of nanomolar to low micromolar range) (Milligan et al., 2022). This suggests that individual components of these nutraceutical products, such as other phytocannabinoids and terpenes, may synergistically further modulate or inhibit Nav channels (Milligan et al., 2022).

In addition to Nav channels, a new study showed that CBD at sub-micromolar concentrations, hyperpolarizes the voltage-dependence of Kv7.2/3. This shift results in an enhancement of the M-current which has a powerful effect on dampening down neuronal excitability and has previously been clinically exploited by effective drugs such as Retigabine (Rundfeldt and Netzer, 2000). This effect may be a key contribution to the anticonvulsive and proposed analgesic activities of CBD, independently of other ion channel modulating effects (Zhang et al., 2022).

## Cannabigerol—A potentially promising avenue for pain treatment via sodium channels

The role of Nav1.7 in the pain pathway is well-established (Dib-Hajj et al., 2013). Many gain- and loss-of-function mutations in Nav1.7 have been identified. Hyperexcitability in this channel has been shown to elicit several pain syndromes (Dib-Hajj et al., 2005, 2013; Fertleman et al., 2006), whilst hypoexcitability in this channel is linked with complete insensitivity to pain, which is not accompanied with any cognitive, cardiac, or motor defects (Cox et al., 2006; Goldberg et al., 2007). These findings highlight the importance of Nav1.7 as an excellent target for pain therapy; however, the efforts that have gone into developing small molecules for Nav1.7 inhibition have thus far been unsuccessful. This lack of success has been attributed to problems in achieving optimum channel occupancy and, thus, problems in effective target engagement (Bankar et al., 2018). To get around this problem, *in vivo* treatments with many folds above IC_50_ could be utilized, but at these high dosages, this would cause unwanted side effects.

A potential advantage of highly hydrophobic compounds like cannabinoids is that they might more readily get absorbed into the lipid dense neuronal tissues and nerve membranes. This would require the mode of administration of the compound to reduce exposure in the central nervous system and increase the probability of distribution into the peripheral nerves, to avoid off target effects in the CNS: central nervous system. If the compound also had either structural or functional selectivity for Nav1.7, then efficacy may be achievable. Whilst structural selectivity would refer to a unique amino acid sequence or motif that is present in one channel (Catterall and Swanson, 2015), but not others, functional selectivity refers to the ligand’s (in this case CBG) increased affinity for the channel that occupies one particular state within its local environment (Kuo and Bean, 1994). As the resting membrane potential of sensory dorsal root ganglion (DRG) neurons is considerably more depolarized than the availability voltage-dependence of Nav1.7 (relative to other sensory Nav channels), Nav1.7 in these neurons accumulates a lot more inactivation than other Nav channels (Amir et al., 1999; Ghovanloo et al., 2022a). Thus, the state-dependent drug that has a higher apparent potency for the inactivated states of these channels, would be predicted to more effectively inhibit Nav1.7 in DRG neurons than the other Nav channels.

CBD and CBG are both highly hydrophobic compounds with very high distribution coefficients for the hydrophobic phase. In fact, CBG (ChEMBL-calculated-LogD = 7.04) is even more hydrophobic than CBD (ChEMBL-calculated-LogD = 6.60), which suggests that CBG may have a higher propensity to enter and remain within the lipid membranes. However, neither compound has much structural selectivity for Nav channels. As noted above, CBD has been shown to bind at the Nav channel pore, which is a highly conserved region of the channel (Catterall and Swanson, 2015; Ghovanloo and Ruben, 2021). Thus, like the other classic pore blockers that bind this region (Hille, 1977; Bean et al., 1983; Gamal El-Din et al., 2018), CBD is not structurally selective. Although CBG has not been functionally tested for its binding site on the Nav channel, it likely interacts within the same site at the pore-fenestration interface.

Because CBG was implicated as an analgesic (Evans, 1991; Mammana et al., 2019), we investigated its effects on Nav channels. We determined that CBG is also a moderately state-dependent Nav channel inhibitor, and shares (CBG is slightly less potent than CBD) many of the same features in its modulation of Nav channels with CBD (Ghovanloo et al., 2022a). We found that CBG does not alter the voltage-dependence of activation or alter open-state inactivation, it hyperpolarizes inactivation curves, slows recovery from inactivation (with this effect becoming more pronounced as the channels enter deeper inactivated states, e.g., slow inactivation), accelerates onset of closed-state fast inactivation, and reduces spontaneous firing of DRG neurons. Importantly, we found that CBG inhibits total channel conductance more potently than it stabilizes the inactivated state of channels (inactivation shift potency = 13.3 ± 1.0 µM, G_max_ inhibition potency=3.4 ± 1.0 µM) (Ghovanloo et al., 2022a) (Figure 1B). As Nav1.7 is known as the threshold channel for peripheral firing (Dib-Hajj et al., 2013), these results suggest that CBG may be more effective in preventing pain episodes from initiating. Given that poly-pharmacological compounds will display more promiscuity in their interactions with various targets at higher concentrations (hence toxicity), the best therapeutic window for CBG’s potential therapeutic efficacy will occur by taking advantage of the Nav channel G_max_ block at the low to sub-micromolar concentrations where CBG’s hyperpolarization of inactivation would be physiologically inconsequential (Ghovanloo et al., 2022a). Finally, the noted mechanism could, in principle, work in concert with CBG’s CB-dependent (without psycho-activity) pathways to target pain.

We suggest that the development of CBD/CBG as Nav channel-targeting drugs may be achievable *via* exploring various modes of administration. For instance, with respect to pain, if the compound could be localized to the nociceptors, then given the physicochemical properties of the compound, along with local resting membrane potential and the availability voltage-dependences of the local Nav channels, the channel that is most inactivated would be most modulated (as noted above, Nav1.7 (Ghovanloo et al., 2018; 2022a)). We also note that the concentrations at which CBD targets slow inactivation of Nav1.8 and Kv7.2/3, on the low end are comparable (Zhang and Bean, 2021; Zhang et al., 2022). Studies suggest that in the case of CBD, mode of administration can substantially alter pharmacokinetics and bioavailability, and hence tissue distribution of the drug (e.g., local administration to work alongside compound hydrophobicity to accumulate in tissues with high lipid content (Ghovanloo et al., 2022a)) (Millar et al., 2018; Lim et al., 2020); however, even then, the drug effect on phenotype would not likely be able to be attributed to activity on a single target (e.g., Na or K currents, TRP channels, etc.), but we propose that Nav channels are likely an important part of the pleiotropic pharmacology of CBD. These considerations would be critical in translation of using these compounds from the bench to the bedside.

## Concluding remarks

CBD and CBG, and indeed other cannabinoids and terpenes are intriguing molecules with highly complex pharmacological profiles. Despite enormous progress in recent years, the precise mechanism of clinical efficacy remains unknown. The Nav channel family is vital to nervous system signalling, and it is likely an important receptor for these molecules. Future investigations into the intricate interactions between Nav channels (and other receptors) and cannabinoids will facilitate unravelling how cannabinoids impart their effects on physiology, which could aid the identification of novel therapeutics for various disorders of neuronal excitability.
